# Effects of heating on bioceramic sealers: a scoping review of chemo-physical properties and clinical implications

**DOI:** 10.1007/s10266-024-00969-3

**Published:** 2024-07-08

**Authors:** Giusy Rita Maria La Rosa, Francesco Saverio Canova, Maria Laura Leotta, Eugenio Pedullà

**Affiliations:** Department of General Surgery and Medical-Surgical Specialties, University of Catania, Catania, Italy

**Keywords:** Bioceramic, Endodontics, Scoping review, Temperature, Warm vertical compaction

## Abstract

This scoping review aims to summarize current research to assess the impact of heating on the chemo-physical properties of bioceramic sealers. Following the Preferred Reporting Items for Systematic Reviews and Meta-Analyses (PRISMA) Extension for Scoping Reviews guidelines, a comprehensive literature search was conducted in April 2024 across PubMed and Scopus databases. Inclusion criteria referred to all study types evaluating the effect of heating temperature on bioceramic sealers' properties, with no language or time restrictions applied. Studies were independently screened by two reviewers, and relevant data were extracted and synthesized qualitatively. Out of 91 initially identified studies, 19 met the eligibility criteria for inclusion in the qualitative synthesis. The selected studies ranged from 2014 to 2024 and comprised laboratory-based investigations. Various bioceramic sealers, including EndoSequence BC Sealer, BioRoot RCS, and TotalFill BC, were analyzed across different heating techniques. The findings revealed divergent responses of bioceramic sealers to heat, with some demonstrating stability while others exhibited alterations in properties such as flow, setting time, and chemical composition. The impact of heat on bioceramic sealers depends on the sealer’s composition and laboratory setting. While some sealers are affected significantly, others remain stable. Clinicians should carefully consider these factors when bioceramic sealers are used with warm obturation techniques, but caution is needed as real-world conditions may vary. Integrating laboratory results with clinical evidence is essential for improving treatment efficacy and patient care.

## Introduction

The primary objective of endodontic therapy is to chemo-mechanically clean the infected root canal system and achieve complete obturation with filling materials to prevent bacterial infiltration into the periapical tissues [1]. Over the years, numerous materials and techniques have been used to seal the endodontic system effectively.

A good sealing material should possess several characteristics, including ease of introduction into the canals, dimensional stability, ability to seal the apex, radiopacity, bacteriostatic properties, and biocompatibility, without causing tissue irritation [2]. However, no single material can fulfill all these criteria perfectly. Therefore, root canal sealers are used in conjunction with filling materials, playing a crucial role in achieving a successful root canal treatment [3].

The main objectives of sealers are to fill voids, gaps, lateral canals, and accessory apical foramina, establish a bond between the obturation material and dentin, and act as a lubricant to favor the positioning of the filling core [4]. Root canal sealers have been extensively studied and classified based on their chemical composition, including zinc oxide eugenol, epoxy-resin based such as AH Plus (Dentsply, Konstanz, Germany) [1], calcium hydroxide, glass ionomer, resin, silicone, and the recently introduced bioceramic sealers (BCs) [4].

Bioceramic sealers represent an advancement in endodontic materials, characterized by their biocompatibility and increasing popularity in clinical practice. These premixed cements are based on tricalcium silicate [5]. Calcium-silicate-based materials exhibit high biocompatibility and bioactivity, capable of forming bonds with pulp or periodontal tissue and stimulating hydroxyapatite production, rendering them osteoconductive and antibacterial [6]. Their chemical properties, including hydrophilicity and radiopacity, along with their ability to expand slightly over time and good flowability, make them suitable for various endodontic procedures [7]. The introduction of BCs has revolutionized obturation techniques, with a shift towards a higher proportion of sealer to gutta-percha, enhancing the biological and antibacterial effects of the treatment [8].

While various obturation techniques have been used with BCs, the single cone technique (SC) has shown promising results. Some studies have assessed the performance of bioceramic sealers using warm obturation techniques [9, 10]. However, the application of heat during warm vertical compaction (WVC) may alter the chemo-physical properties of calcium-silicate-based materials, affecting their flow and setting time, thereby potentially impacting the effectiveness of root canal sealing and treatment outcomes [7].

Currently, no comprehensive reviews have systematically evaluated the current literature to investigate the impact of heating on chemo-physical properties of BCs. Thus, the objective of this scoping review is to synthesize current studies to determine whether the heating adversely affects the chemo-physical properties of bio-ceramic sealers.

## Materials and methods

This scoping review was reported according to the Preferred Reporting Items for Systematic Reviews and Meta-Analyses (PRISMA) Extension for Scoping Reviews [11] and answered the question: “What is the effect of heating on chemo-physical properties of bioceramic sealers?”.

### Inclusion criteria

All types of studies (i.e., human, animal and in vitro studies) that evaluated the impact of heating temperature on chemo-physical properties of BCs were included. Studies on obturation techniques were included only if they contained data on the effect of temperatures on chemo-physical properties of BCs. This criterion ensured that the review focused specifically on understanding how heating impacts the characteristics of BCs used in endodontic procedures. No language and time restrictions were applied.

### Exclusion criteria

Case reports and case series, systematic and narrative reviews, abstract conference, brief communications, and letters to the editor were not considered.

### Primary and secondary searches

In April 2024, an extensive search of the literature was conducted. Two reviewers independently conducted the search on PubMed and Scopus databases using MESH terms and keywords “bioceramic*”, “calcium silicate”, “heat*”, “temperature*”, and “endodontic*”. Additionally, the references of the included articles were examined for other potentially relevant studies. Key peer-reviewed scientific journals in endodontic research as well as miscellaneous journals including the *Journal of Endodontics*, *International Endodontic Journal*, *Australian Endodontic Journal*, *European Endodontic Journal*, *Journal of Dentistry*, *Dental Materials*, *BMC Oral Health*, *Clinical Oral Investigations,* and *Odontology* were manually searched. Titles and abstracts of published articles were screened by two independent reviewers to ensure they met the inclusion criteria, and any duplicates were eliminated. The references were managed by EndNote (EndNote 21; Thomson Reuters, New York, NY, USA).

### Studies’ selection

Abstracts with incomplete information were provisionally included and subsequently assessed independently through full-text reading by the same two reviewers. Any discrepancies regarding inclusion were addressed through discussion until consensus was reached; no involvement of a third examiner was required. Articles meeting the inclusion criteria were chosen for qualitative synthesis.

### Extraction and synthesis of relevant data

Relevant data were extracted utilizing a standardized data form tailored specifically for this task. The form was designed to record and tabulate the following items: author & year, country, sample, bioceramic, sealer, heating technique, control, methodology, outcomes and main findings. The qualitative synthesis focused on the modifications in chemo-physical properties of BCs after heating and their clinical implications.

## Results

**Fig. 1:**
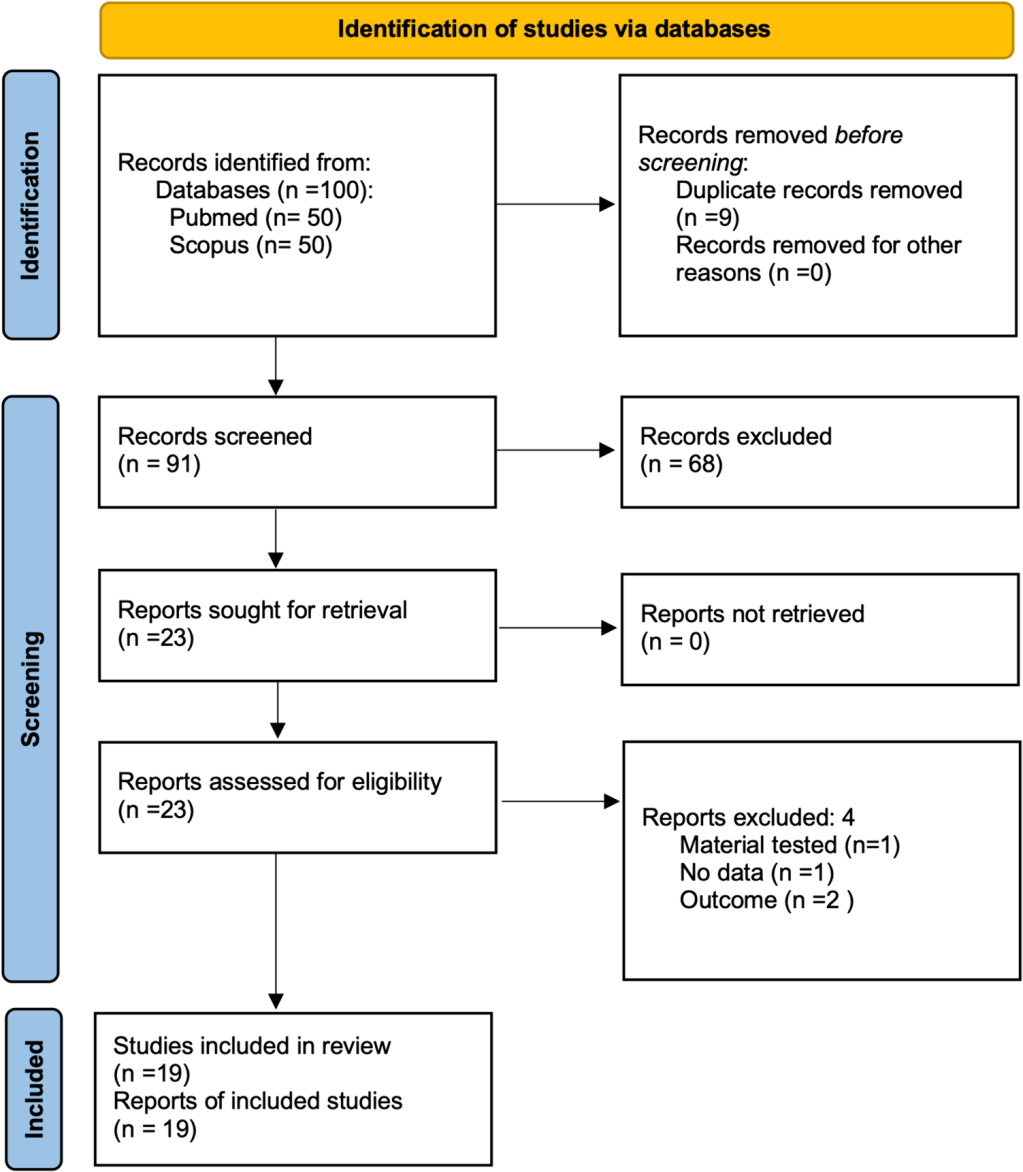
Flow chart of the review process

The study selection is shown in Fig. 1, according to PRISMA 2020 for scoping reviews. A total of 91 potentially relevant studies were retrieved. Among these, 72 studies did not meet the inclusion criteria, so they were removed. Finally, 19 studies satisfied the inclusion criteria for qualitative synthesis [1, 7–9, 12–26]. The main features of the selected studies are reported in Table 1.

**Table 1:** General characteristics of the included studies

Author & year	Country	Sample	Bioceramic sealer	Heating technique	Control	Methodology	Outcomes	Main findings
Alberdi Koki et al., (2023) [21]	Argentina	Lower premolars (*n* = 7/group)	Bio C Sealer (Angelus, Brazil) CeraSeal (MetaBiomed, Korea) BioRoot RCS, (Septodont, France)	Warm vertical compactionand a carrier-based technique, Guttacore using a Therma-Prep oven (DentsplyMaillefer, Ballaigues, Switzerland)	NA	Universal testing machine Light microscope	Push-out bond strenght	The heat produced by the obturation methods did not influence the bonding of the examined bioceramic sealers to the dentinal surface
Al-Hiyasat and Alfirjani. (2019) [19]	Jordan	Human maxillary first premolars (*n* = 10/group)	TotalFill BC Sealer (FKG Dentaire SA, La-Chaux-de-fonds, Switzerland)	Heated plugger and Calamus (Dentsply Tulsa) at 200 °C	AH Plus sealer (Dentsply DeTrey, Konstanz, Germany)	Universal testing machine Optical microscope	Push-out bond strength	The push-out bond strength of TotalFill BC sealer was higher that of AH Plus sealer, and while the WVC significantly impacted AH Plus sealer, it did not affect TotalFill BC sealer
Aksel et al., (2021) [14]	USA	Sealer samples (*n* = 3–9/group)	BioRoot RCS (Septodont, Saint-Maur-desFossés, France) Endosequence BC Sealer (Brasseler, Savannah, GA) Endosequence BC Sealer HiFlow (Brasseler)	Incubator at 37 °C and laboratory oven (Model 40GC, Quincy Lab, Chicago, IL, USA) at 200 °C	AH Plus (Dentsply Sirona, York, PA, USA)	TGA FT-IR Universal testing machine Indenter Rheometer	Changes in chemical composition Setting time, viscosity,flow	WVC obturation techniques appear to be compatible with HiFlow, Endosequence, and AH Plus sealers. Heat application resulted in minor alterations in their physical and chemical characteristics. In contrast, BioRoot RCS exhibited a substantial weight loss, increased viscosity, and reduced flowability following heat exposure
Antunes et al., (2021) [22]	Brazil	Sealer samples (*n* = 6/group)	BioRoot RCS (Septodont) EndoSequence BC Sealer HiFlow (Brasseler) Bio-C Sealer (Angelus)	Oven at 37 °C and 100 °C	AH Plus (Dentsply Sirona, York, PA, USA)	Raman spectroscopy FT-IR SEM–EDS Digital caliper Moist and dry methods Digital radiography Digital pH meter	Changes in structure and chemical composition Flow, setting time, solubility, radiopacity, pH	Heating had an impact on the calcium silicate-based sealers included in the study. These sealers exhibited high solubility, which is considered a limitation for their clinical application. Among the tested sealers, AH Plus was the only sealer found to remain stable following heating
Atmeh and AlShwaimi.(2017) [12]	Saudi Arabia	Sealersamples (*n* = 5/group)	BioRoot RCS (Septodont)	Electronic oven (Blue M, Blue Island, IL): T200°_30s, T200°_60s, T250°_30s, T250°_60s	AH Plus (Dentsply, Konstanz, Germany)	Raman spectroscopy TGA	Changes in the chemical composition and the effect of heat on mass change where sealers were heated	The duration and temperature of heat application can potentially influence the chemical composition of epoxy resin sealers. Given the importance of endodontic sealer compatibility, it is crucial to consider both the endodontic sealer's compatibility and the duration of heat application, particularly when employing WVC techniques
Camilleri. (2015) [15]	Malta	Sealer samples (*n* = 3/group)	MTA Fillapex (Angelus) Septodont (Saint Maurdes-Fosses, France)	Climatic chamber at 37 °C and System B (Analytic Technology, Redmond, WA) at 100 °C	Apexit Plus (Ivoclar, Schaan, Lichtenstein) AH Plus (Dentsply International, Addlestone, UK)	SEM–EDS XRD FT-IR Modified Vicat apparatus Micrometer	Changes in structure and chemical composition Setting time, flow, film thickness	Based on the findings, it appears that the Septodont sealer is well-suited for obturations utilizing cold laterally condensed gutta-percha, whereas MTA Fillapex and Apexit Plus are recommended for warm gutta-percha obturation techniques
Chavarria-Bolanos et al., (2022) [24]	Costa-Rica	Sealer samples (*n* = 6/group)	MTA-Fillapex (Angelus)	DSC unit Q200 (TA Instruments, New Castle, DE, USA) at 180ºC, 200ºC, and 230ºC	AH Plus/ TopSeal (Dentsply Sirona, Konstanz, Germany) Adseal META (Biomed, Chungcheongbuk-do, Korea) RoekoSeal (Coltene/Whaledent, Cuyahoga Falls, OH, USA) GuttaFlow 2 (Coltene/Whaledent) Roeko GuttaFlow BioSeal(Coltene/Whaledent) EndoRez (Ultradent, South Jordan, UT, USA)	DSC SEM–EDS TGA	Changes in structure, chemical composition and thermodynamic profile	Elevated temperature exposure adversely affected the properties of five sealers (Adseal, MTA-Fillapex, RoekoSeal, GuttaFlow 2, and EndoRez)
Chen et al., (2020) [17]	Canada	Sealer samples (*n* = 6/group) Culture of human periodontal ligament fibroblasts	EndoSequence BC Sealer (Brasseler) EndoSequence BC Sealer HiFlow (Brasseler)	Storage at 37 °C and oven (Sheldon Manufacturing Inc, Cornelius, OR) at 100 °C	NA	Indentation technique Digital caliper Universal test machine Microhardness tester Digital radiography Analitical balance Rheometer FT-IR	Cell viability, setting time, flow, film thickness, microhardness, radiopacity, solubility, viscosity and shear rate	HiFlow exhibited superior performance in terms of flow/viscosity and film thickness, particularly under elevated temperatures typically generated by the commonly used WVC technique
DeLong et al., (2015) [18]	USA	Human single-rooted teeth (*n* = 10/group)	EndoSequence BC Sealer (Brasseler) MTA Plus (Avalon Biomed Inc, Bradenton, FL)	System B; (SybronEndo, Orange, CA) at 200 °C	AH Plus sealer (Dentsply DeTrey, Konstanz, Germany)	Universal testing machine Operating microscope	Push-out bond strength	The continuous wave technique resulted in decreased bond strength for EndoSequence BC Sealer and MTA Plus, indicating that it is not advisable for use with these sealers
Dewi et al., (2022) [7]	Thailand	Human mandibular premolars (*n* = 10/group)	TotalFill BC Sealer (FKG Dentaire SA) EndoSequence BC Sealer HiFlow (Brasseler)	Heat carrier tip (SuperEndo Alpha; B&L Biotech, Gyeonggi-do, South Korea) at 200 °C	AH Plus sealer (Dentsply Sirona, Charlotte, NC, USA)	Universal testing machine Inverted phasecontrast microscope	Push-out bond strength	Heat-based obturation technique (WVC) did not impact the push-out bond strength of the TotalFill BC sealer and EndoSequence BC Sealer HiFlow. Both pre-mixed sealers had higher push-out bond strength than AH Plus
Donnermeyer et al., (2021) [8]	Germany	Sealer samples (*n* = 2/group)	TotalFill BC Sealer HiFlow (FKG Dentaire) TotalFill BC Sealer (FKG Dentaire) BioRoot RCS (Septodont)	K-type thermocouple (GHM Messtechnik, Regenstauf, Germany) was placed inside the samples, which were heated in a thermo-controlled water bath until the temperatures of 37 ◦C, 47 ◦C, 57 ◦C, 67 ◦C, 77 ◦C, 87 ◦C and 97 ◦C	NA	FT-IR A cylindrical indenter Universal testing machine Digital caliper	Changes in chemical composition Setting time, film thickness, flow	Thermal treatment did not induce significant chemical changes across all temperature ranges, although the physical properties of BioRoot RCS were compromised by heating. TotalFill BC Sealer HiFlow and TotalFill BC Sealer appear to be well-suited for WVC techniques
Hadis and Camilleri. (2020) [20]	UK	Sealer samples (*n* = 3/group)	TotalFill HiFlow BC Sealer (FKG Dentaire) TotalFill (FKG Dentaire)	Heat carriers: E&Q Master (Meta Biomed, Chalfont, PA, USA) at 180°, 230° System-B (Orange, CA, USA) at 200°	NA	SEM–EDS XRD TGA–DSC	Surface and chemical sealer characterization after heating and suitability at clinical temperature	Both sealers were resistant to heat and showed identical chemistries except for the organic component’s variations. TotalFill BC sealer is suitable for use with WVC technique as it is cheaper and as effective as the HiFlow
Ito et al., (2024) [25]	Japan	Sealer samples (*n* = 8/group)	EndoSequence BC Sealer (Brasseler) AH Plus Bioceramic Sealer (Dentsply Sirona, Charlotte, NC, USA)	Storage at 37 °C and oven (007S, KDF, Kyoto, Japan) at 100 °C	AH Plus Jet (Dentsply Sirona, Charlotte, NC, USA) Pulp Canal Sealer (Kerr, Brea, CA, USA; PCS)	SEM–EDS Gilmore-type metric indenter Micrometer	Setting time, flow, film thickness	The detected changes in physical properties can negatively impact the performance of premixed calcium silicate-containing sealers, particularly AH Plus Bioceramic Sealer, when WVC is employed
Karam et al., (2024) [23]	Lebanon	Sealer samples (*n* = 16/group)	AH Plus Bioceramic sealer (Dentsply Sirona, York, PA, USA) TotalFill BC sealer (FKG Dentaire) One-fil Bioceramic sealer (MEDI- CLUS, Cheongju, Korea) K-Biocer sealer (Prototype)	Oven at 37 °C, 60 °C, and 200 °C for 30 s	Any-seal® resin-based sealer (MEDICLUS, Cheongju, Korea)	SEM–EDS Micrometer	Surface characterization Setting time, film thickness, flow, dimensional change	Heat caused surface deformations in all the tested sealers EDS analysis detected heat-induced chemical and alterations in all examined sealers.
Nomura et al., (2023) [26]	Brazil	Sealer samples (*n* = 3–6/group)	Endosequence BC sealer, (Brasseler) BioRoot RCS, (Septodont) Bio-C Sealer (Angelus)	Incubator at 37 °C and oven (ECB2, Odontobrás, Ribeirão Preto, SP, Brazil) at 100 °C	AH Plus (Dentsply Sirona, York, PA)	SEM–EDS Indentation technique with a Gilmore-type metric indenter Digital caliper pHmeter	Changes in structure, chemical composition Setting time, flow, sealer solubility and dimensional change	Heat application was associated with the decrease in the EndoSequence BC sealer setting time. Also, heating promoted significant surface changes in all sealers
Qu et al., (2016) [9]	China	Sealer samples (*n* = 10/group)	iRoot SP (Innovative Bioceramix, Vancouver, Canada)	Incubator at 37 °C and an electric heating oven (Tianjin Zhonghuan Co, Ltd, Tianjin, China) at 140 °C	Zinc oxide–eugenol (ZOE) AH Plus (Dentsply International, York, PA) RoekoSeal (Roeko/Coltene/Whaledent, Langenau, Germany)	Stereoscopic microscope Indentation technique with a Gilmore-type metric indenter Micrometer	Setting time, flow and porosity	Warm vertical compaction affected the setting time, flow, and porosity of the sealers under examination. RoekoSeal and iRoot SP sealers notably experienced a substantial decrease in setting time and flow at elevated temperatures
Viapiana et al., (2014) [13]	Malta	Human maxillary canines (*n* = 4/group) and sealer samples (*n* = 8/group)	MTA Fillapex (Angelus Dental Solutions)	System B (Analytic Technology, Redmond, WA) at 200 °C	AH Plus (Dentsply International, Addlestone, UK) Prototype sealer based on Portland cement (Araraquara Dental School, São Paulo State University, Brazil)	FT-IR Indentation technique with a modified Vicat needle Compressive strength testing	Changes in chemical composition and heat dissipated at the external root surface Setting time and the sealer’s strength	Environmental conditions played a role in heat dissipation during the continuous wave of condensation obturation technique, with root canal sealers exhibiting varying conductive or isolating properties. While the temperature had no discernible effect on MTA Fillapex, Pulp Canal Sealer, and the prototype sealer, it adversely impacted the properties of the AH Plus sealer
Viapiana et al., (2015) [16]	Malta	Human maxillary canines (*n* = 4/group) and sealer samples	MTA Plus mixed with antiwashout gel (Prevest Denpro, Jammu, India for Avalon Biomed Inc. Bradenton, FL, USA) TCS-20-Z polymer (University of Malta)	System B (Analytic Technology, Redmond, WA) at 200 °C	AH Plus (Dentsply International Addlestone, UK) TCS-20-Z epoxy (University of Malta)	SEM–EDS XRD FT-IR Light microscope	Surface characterization Physical and chemical changes and heat dissipated at the external root surface	The sealers effectively reduced the heat generated on the external root surfaces during the heating phase. AH Plus exhibited alterations to its chemical structure following exposure to heat, whereas the other sealers remained unaffected
Yamauchi et al., (2020) [1]	Japan	Sealer samples (*n* = 5–8/group)	Endoseal MTA (Maruchi, Wonju, Republic of Korea) Well-Root ST (Vericom, Gangwon-Do, Republic of Korea) EndoSequence BC Sealer (Brasseler) EndoSequence BC Sealer HiFlow (Brasseler)	Incubator at 37 °C and an oven (007S, KDF, Kyoto, Japan) at 100 °C	AH Plus (DeTrey Dentsply, Konstanz, Germany	SEM–EDS Gilmore-type metric indenter Microcaliper pH meter	Surface characterization Setting time, flow, film thickness, and pH	When subjected to heating, calcium silicate-based root canal sealers exhibited accelerated setting time, decreased flow, and increased film thickness at 100 °C for 1 min. However, the magnitude of these alterations varied among the sealers. These changes in physical properties may negatively impact the quality of root canal filling when calcium silicate-based sealers are utilized for the WVC technique.

*DSC* Differential scanning calorimetry*, EDS* Energy-dispersive spectroscopy*, FT-IR* Fourier transform infrared spectroscopy*, NA* Not Applicable*; SEM* scanning electron microscope*, TGA* Thermogravimetric Analysis*, XRD* X-ray diffraction analysis*, WVC* warm vertical compaction

### Main features of the selected studies

The selected studies were published from 2014 to 2024. Three were conducted in Malta [13, 15, 16]. two in the USA [14, 18], two in Japan [1, 25] and other two in Brazil [22, 26]. The remaining were carried out in Argentina, Brazil, China, Costa-Rica, Germany, Jordan, Lebanon, Saudi Arabia, Thailand and the UK [7–9, 12, 19–21, 23, 24]. All studies were designed as laboratory (ie, in vitro) studies.

Overall, sealer’s standardized specimens were the most common type of samples investigated. Three studies performed the tests on extracted human teeth [7, 13, 16, 18, 19, 21], using bioceramic cements in association with the main warm root canal obturation techniques, such as warm vertical compaction, and a carrier-based technique.

AH Plus (Dentsply, Konstanz, Germany) is the most common epoxy resin-based sealer that has been used as a control group in almost 68% of the included studies [1, 7, 9, 12, 14–16, 18, 19, 22, 24–26]. The most frequent bioceramic sealers investigated were: EndoSequence BC Sealer (Brasseler, Savannah, GA, USA) [1, 14, 17, 18, 25, 26], BioRoot RCS (Septodont, Saint Maur Des Fosses, France) [8, 12, 14, 21, 22, 26], EndoSequence BC Sealer HiFlow (Brasseler) [1, 7, 14, 17, 22], and TotalFill BC sealer (FKG Dentaire, La Chaux des Fonds, Switzerland) [8, 19, 23].

Nine studies assessed the sealer surface’s characterization by scanning electron microscopy and energy-dispersive spectroscopy (SEM–EDS) [1, 15, 16, 20, 22–26], seven the chemical modifications before and after the application of a heating source by Fourier Transform Infrared Spectroscopy (FT-IR) [8, 13–17, 22] and twelve investigated the physical properties including setting time, flow and film thickness [1, 8, 9, 13–17, 22, 23, 25, 26].

### Main findings

Overall, increased temperature-induced changes on the chemo-physical properties of BCs investigated, which depended on the type of the product tested.

More specifically, Endosequence BC, Endosequence BC HiFlow and TotalFill HiFlow BC sealers seem to be suitable for warm obturation techniques [1, 14, 17, 20]. They possess similar setting time, microhardness, solubility, chemical composition, and cytotoxicity, and when exposed to heat, they did not show an alteration to the chemical structure. Endosequence BC HiFlow exhibited higher radiopacity and demonstrated superior flow/viscosity characteristics and film thickness, particularly under elevated temperatures typically associated with the commonly employed hot vertical compaction techniques [1, 14, 17]. The same studies reported that the application of heating to AH Plus cement caused faster degradation of the N–H bond resulting in a faster setting reaction [1, 14]. Similarly, heat treatment did not affect the composition of TotalFill BC and TotalFill BC HiFlow calcium silicate sealants [8, 20]. Although TotalFill BC HiFlow showed higher flow and higher setting time, the changes did not fall below clinically relevant thresholds, and the film thickness appeared to be unaffected by temperature [8]. In contrast, other studies reported that bioceramic cements, including Endosequence BC, were negatively affected by the temperature increasing [8, 12, 18, 22–26]. In particular, heat negatively impacted BioRoot RCS which had a significant amount of weight loss, increased viscosity, and lowered flowability after heat application [8, 26].

To evaluate the bond strength of bioceramic sealers to gutta-percha and root canal walls, after obturation, specimens were sectioned into 1-mm root slices, subjected to a push-out bond test using a universal testing machine and observed under a microscope [7, 18, 19, 21]. The results showed that heat-based obturation technique did not impact the bond strength of Endosequence BC HiFlow [7], TotalFill BC [7, 19], Bio C Sealer, CeraSeal and BioRoot [21] while the CW technique decreased the bond strength of EndoSequence BC Sealer and MTA Plus [18].

## Discussion

Tricalcium silicate-based cement represent a promising option for root canal filling due to their superior sealing properties [27] and excellent biocompatibility [28–30]. The choice between WVC and cold condensation techniques for gutta-percha adaptation in root canals can influence the quality of obturation, with the former allowing for a more uniform and dense filling [1]. However, concerns exist regarding the effects of heat on the chemo-physical properties of bioceramic sealants [31]. Due to the heterogeneity of the available studies in terms of materials tested and methodological conditions, we decided to perform a scoping review for mapping the current evidence and identify knowledge gaps [32, 33].

### Experimental approach

Studies have investigated the impact of heat on bioceramic sealants by subjecting sealer’s samples to different temperatures. The samples were prepared in accordance with the manufacturer's instructions, compacted, and heated. Validated analysis techniques such as FT-IR and SEM–EDS were employed to assess changes in chemical structure and characterize the sealer’s surface. In addition, warm obturation techniques, operating typically between 200 ℃ and 250 ℃ for 10 s, have been studied to understand their effects on sealers. This methodological approach allowed for the necessary control of variables required for in vitro testing. However, it does not account for all the clinical variables that could influence the dispersion of the applied heat. The push-out test is a popular method for assessing the clinical performance of sealants, involving the application of vertical force using a stainless-steel plunger on root slices to measure detachment resistance. Factors like sample thickness, piston diameter, and orientation can influence detachment resistance values in this test [34, 35].

### Research insights from in vitro studies

Heating may significantly impact the properties and clinical behavior of endodontic bioceramic sealers. Exposure to high temperatures can lead to the thermal degradation of organic components which can alter the sealer's flowability, setting time, and film thickness [22, 25]. Additionally, heat can influence the solubility of bioceramic sealers, with calcium silicate-based sealers exhibiting increased solubility when exposed to high temperatures, potentially affecting their ability to maintain a seal in the root canal [23]. On the other hand, some endodontic bioceramic sealers are not significantly affected by heating due to their inherent properties and composition [1, 8, 14, 17, 20]. For example, the EndoSequence BC sealer showed decreased setting time and increased solubility when heated, but no significant changes in flowability, degree of conversion, or pH were observed [26]. Additionally, the physical and chemical properties of bioceramic sealers can be tailored to withstand heat-induced changes, ensuring their stability and sealing ability even when heated. The EndoSequence BC Sealer HiFlow contains the same components as the previously available BC sealers, consisting of tricalcium silicate, dicalcium silicate, and calcium phosphates. According to the manufacturer, this new HiFlow material has greater flowability due to its lower viscosity, resulting in more suitable for heat-based obturation techniques. Additionally, it can withstand high temperatures, which is beneficial for these techniques [7, 14]. However, modifications in physical properties have been reported also for this sealer after heating [1, 22].

The different results among studies can mainly be attributed to differing methodological conditions, particularly the exposure temperature (ie, 100 ℃ vs 200 ℃) and duration of heat exposure [14, 17]. These factors are confirmed to be two of the primary variables, along with the composition of the bioceramic tested, to be considered in evaluating the effects of heat on the chemical and physical properties of the bioceramic. Moreover, the use of some devices in continuous mode may induce elevated temperature that may overcome the safety levels, compromising the integrity of obturation materials [36].

### Limitations

The key limitations of the studies on the impact of heating on the chemo-physical properties of bioceramic sealants should be acknowledged. The nature of the outcome necessitates a laboratory setting to investigate the effects of heating, but the results may not directly translate to the clinic. These studies may not fully reproduce the characteristics of the oral environment, as they often do not consider factors such as the alveolar bone and periodontal ligament in which the dental element is located. Additionally, limitations are imposed by the observation time at which measurements were conducted. While laboratory results are valuable for gaining insights into the behavior of bioceramic sealers, observational studies with long-term follow-up are appropriate to assess the clinical performance and impact of using these sealers with warm techniques on clinical outcomes [37, 38].

### Clinical implications

The impact of heating on the chemo-physical properties of bioceramic sealers varies by product. Endosequence BC and Endosequence BC HiFlow may exhibit suitable characteristics for hot obturation techniques. TotalFill BC and TotalFill BC HiFlow showed minor changes in flow and setting time, while BioRoot RCS displayed significant weight loss, increased viscosity, and reduced flowability post-heating in most of the studies included. The bond strength of bioceramic sealers to gutta-percha and dentin was influenced by the obturation technique, with Endosequence BC, TotalFill BC, and others demonstrating strong push-out bonding in warm obturation. These findings highlight the importance of considering the specific material’s characteristics and heating conditions when selecting bioceramic sealants in endodontic procedures.

## Conclusions

The effects of heat on the chemical and physical properties of bioceramic sealers seem to depend on the particular composition of the bioceramic material and the methodological conditions, such as temperature and exposure time. Although laboratory studies have revealed significant effects of heat on the chemo-physical parameters of bioceramic sealers, some sealers have exhibited acceptable stability and resilience to thermal stress. It is imperative for clinicians to carefully consider the specific characteristics of the bioceramic they intend to use, especially when employing heat-based obturation techniques. While laboratory findings offer valuable insights, caution in clinical applications is necessary as real-world conditions may diverge from experimental settings.

## Data Availability

No new data were generated in this study. Data sharing is not applicable to the current review.
